# How best to provide help to bereaved adolescents: a Delphi consensus study

**DOI:** 10.1186/s12888-021-03591-7

**Published:** 2021-11-23

**Authors:** Anna M Ross, Karolina Krysinska, Debra Rickwood, Jane Pirkis, Karl Andriessen

**Affiliations:** 1 Centre for Mental Health, Melbourne School of Population and Global Health, The University of Melbourne, 207 Bouverie St., Carlton, VIC 3053 Australia; 2 Faculty of Health, University of Canberra, Bruce ACT, Canberra, 2617 Australia

**Keywords:** Adolescents, Bereavement, Grief, Guidelines, Help-seeking, Suicide

## Abstract

**Background:**

Many adolescents struggle with their grief and mental health issues after the death of a close person, such as a family member or a friend. Given the potentially devastating impact of the loss on the adolescent and their family, professional help can be warranted. However, little is known about how to best help these adolescents. This study aimed to address this gap by determining what help professionals (i.e., counselors) should provide to bereaved adolescents.

**Methods:**

The Delphi method was used to achieve consensus regarding the importance of statements that describe actions a helping professional can take to help a bereaved adolescent. Statements were compiled through a systematic search of the scientific and grey literature, and reviewing interview data from a recent related research study with bereaved adolescents, parents and counselors. An expert panel (*N* = 49) comprising 16 adolescents, 14 parents and 19 helping professionals, rated each statement. Statements that were endorsed by at least 80% of panellists were considered consensus recommendations.

**Results:**

Panellists endorsed 130 out of 190 statements as appropriate actions. These included help for a bereaved adolescent being offered on an ongoing basis, with support to be provided flexibly to meet individual adolescent needs and to acknowledge the agency of the adolescent. Support after a loss by suicide should be tailored to address specific suicide-related issues. Parents of bereaved adolescents should also be offered support so that they are better equipped to help their bereaved adolescent.

**Conclusions:**

This study identified consensus recommendations on how a helping professional might best help bereaved adolescents. It is hoped that these recommendations will guide helping professionals and enhance adolescent grief interventions.

**Supplementary Information:**

The online version contains supplementary material available at 10.1186/s12888-021-03591-7.

## Background

Many adolescents experience the death of a close person such as a family member or a friend; however, little is known about how best to help bereaved adolescents who struggle with their loss. According to the literature, about 4% of adolescents in Western countries have lost a parent before the age of 18, and about 7% have lost a parent or a sibling [1, 2]. About 78% of adolescents have lost a relative or a friend before adulthood [3]. Moreover, about 5% of adolescents have lost someone to suicide over a 12-month period and 18% have done so before their adulthood [4].

Bereavement in adolescence is a disruptive event with the potential for devastating impact, both in the short- and long-term [5]. Common grief reactions in bereaved adolescents include crying, feelings of sadness, numbness, guilt and longing, and ruminative thoughts about the deceased person [6]. Compared with non-bereaved adolescents, bereaved adolescents have increased risks of mental health problems, such as anxiety and depression [7]; decreased developmental competencies (e.g., regarding school, work and relationships [5, 8]; and higher rates of death by suicide and other early mortality [9]. While grief processes after any type of death appear to be similar [10], adolescents bereaved by suicide may face stronger feelings of shock, anger, rejection and self-blame than adolescents bereaved by natural causes. They may also experience less social support and increased risk of mental health problems and suicidal behaviour compared to other bereaved adolescents [11, 12].

The literature indicates the importance of the relational context of bereavement in adolescents [13, 14]. In addition to psychological reactions, bereavement in adolescents has a strong impact on their relationships with friends and the family system [15]. Pre-loss factors, such as personal and family history of mental health problems, and post-loss factors, such as the quality of the remaining relationships especially with a parent, affect the impact of the loss in adolescents with regards to their grief, and emotional and behavioural reactions [16]. The accumulation of various types of problems in adolescents bereaved by suicide or other causes of death points to the risk of a developmental cascade (i.e., a recursive process involving grief reactions, mental health and relational problems, and developmental competencies leading to potentially lasting ramifications) [8].

Responding to an apparent need, various types of adolescent grief support have become available, including counseling, support groups and family interventions [17, 18]. Some services are offered to all bereaved adolescents, and other services target specific groups such as those bereaved by suicide. While most services are offered in-person, some are offered online (e.g., online support groups), and others include outreach [6, 18, 19]. Parents are the most important facilitators of help-seeking in bereaved adolescents [20, 21]. However, due to a paucity of intervention studies, little is known about the effectiveness of services offered to bereaved adolescents [22–24]. Nonetheless, evidence suggests that professionally led interventions based on compassionate, supportive and psychoeducational approaches, which also involve a parental component, hold promise in terms of effectiveness for helping bereaved adolescents [18, 25].

While translation of research findings into practice remains challenging, research should be informed by clinical experience, as well as the experiences of bereaved adolescents and families, to be clinically relevant and to address the needs of the bereaved adolescents. Our research with bereaved adolescents and parents revealed that bereaved adolescents value a genuine relationship with a helping professional and want to feel understood [15, 20]. In addition, they want to maintain control over the counseling process and be able to access services when they need it. However, due to a shortage of research with helping professionals, further research involving adolescents and parents, as well as helping professionals and service providers, is needed to examine how their views on helping bereaved adolescents align with each other. Where it is impractical to determine best-practice through experimental research, expert consensus methods can be used to gather practice-based evidence from the experiences of experts to determine best-practice [26].

This study is part of a larger research project examining how to best help bereaved adolescents. Within that larger research project, we conducted a sizeable qualitative study with bereaved adolescents, parents and clinicians, (the APC study, [15]). Findings regarding views of bereaved adolescents and parents on the impact of the death on adolescents, as well as the views of bereaved adolescents on the helpfulness of bereavement support, have been reported elsewhere [15]. This study builds on the APC study findings and aims to investigate what help helping professionals should provide to bereaved adolescents. It takes the form of a Delphi consensus study that includes bereaved adolescents, parents of bereaved adolescents, and helping professionals who work with these adolescents. For the purpose of the Delphi study, we used ‘helping professionals’ as an umbrella term including counsellors, psychotherapists and other clinicians. The study focuses on bereavement after all types of death. However, given the potential adverse outcomes in the context of grief after suicide and the services that are specifically offered to adolescents bereaved by suicide, suicide bereavement is a specific focus of the study.

## Methods

### The Delphi method

The support that helping professionals should provide to bereaved adolescents was determined using the Delphi consensus method, as applied to mental health-related research [26]. This method is effective for establishing an evidence-base when the literature on the topic is lacking. The Delphi method involves three stages: sourcing statements, development of the Delphi survey, and the formation of expert panel who complete the survey over several survey rounds.

#### Sourcing statements

A systematic search of the scientific and grey literature was conducted to source statements about how a helping professional should help a bereaved adolescent. The scientific literature was searched using PsycINFO and Medline databases using both search and index terms including: adolescent, teenage, youth, grief, bereavement, counselling, support, help. An example of the full search terms is included in Appendix A. The search was limited to English language publications, and research published within the past 10 years (from 2011 to the time of the search in July 2020). Statements that described how a helping professional can help a bereaved adolescent were included. Publications that did not have a focus on adolescent bereavement were excluded.

The grey literature was searched using Google search engines of English-speaking countries (Google.com, Google.com.au, Google.co.uk, Google.co.nz, Google.ca), using Google Chrome in incognito mode to avoid potential bias from the search history. Similar combinations of the search terms as those used to search the scientific databases were used to search the Google search engines (see Appendix A for full search string). The first 50 results on each search were screened for relevant content by one researcher (AR). Of the total 250 results screened, eight were determined to be relevant, including websites such as GoodGrief.org.au and the Suicide Call Back Service website, an online information booklet produced by the Government of South Australia, and three free-to-access online book chapters.

In addition, deidentified transcripts from 28 individual and 11 group interviews, with a total of 20 bereaved adolescents, 18 parents/guardians of a bereaved adolescent, and 34 counsellors, conducted in the APC study [15] were reviewed for statements regarding how a helping professional should help a bereaved adolescent. One researcher (AR) read the transcripts and extracted the relevant statements.

#### Survey development

Statements that clearly outlined how a helping professional could provide support to an adolescent were extracted from the relevant literature and transcripts. For example, the statement “adolescents expect clinicians to be trained and qualified to work in the area of death and bereavement” (p.5) was extracted from research findings [20]. The statements that were extracted from the literature and interview transcripts were compiled and drafted into items for the Delphi survey. Two researchers (KA and AR) then examined the items to ensure each contained a separate idea to the other extracted items and that it was within the scope of the helping professional role. Items were also rephrased to improve clarity, ensuring the actions for how a helping professional or bereavement service can support a bereaved adolescent were clearly described. Following this, the revised items were sent out to the wider research team (KK, DR and JP) for further revisions, which included the additions of brief descriptions and examples to help explain terms related to counselling or therapeutic approaches. These items then formed the content of the survey, and were grouped into six sections based on thematic categories: Section 1: Training and resources for helping professionals; Section 2: Approach to providing support; Section 3: Supporting the bereaved adolescent; Section 4: Involving others in supporting the adolescent; Section 5: Bereavement support services; and Section 6: Suicide bereavement specific support.

#### Delphi panel formation

The expert panel comprised three groups with expertise in managing adolescent bereavement: bereaved adolescents, parents and guardians of bereaved adolescents, and helping professionals who provide support to bereaved adolescents. Adolescents could participate if they: i) were aged 16+ years; ii) had experienced the death of a close person, such as a family member or a friend, when they were aged between 12 and 18 years; and iii) had experienced the death between 6 months and 10 years ago. Parents or guardians could participate if they were the parent or guardian of an eligible adolescent. Adolescents and parents/guardians could participate whether or not their own parent or child participated. Eligible helping professionals had a minimum of 5 years of experience in providing professional support to adolescents experiencing bereavement.

In order to recruit these groups, various organisations disseminated the study announcement throughout their networks. These included bereavement organisations (e.g., Jesuit Social Services, Standby Support After Suicide, The Compassionate Friends, Australian Centre for Grief and Bereavement, Anglicare SA, Survivors of Suicide Bereavement Support Association, Dr. Edward Koch Foundation, Feel the Magic, and Sabrina’s Reach 4 life), youth mental health organisations (e.g., headspace, Orygen Youth Health, and ReachOut Australia), and mental health and suicide prevention organisations (e.g., Beyond Blue’s Blue Voices, and Wesley LifeForce). The study was advertised on The University of Melbourne Student Portal, on the Australian Association of Social Workers website, in the Psychotherapy and Counselling Federation of Australia newsletter, and on social media through Twitter, LinkedIn and Facebook. Participants who had taken part in an interview in the APC study and consented to be contacted about future studies about adolescent bereavement, were invited to take part in the Delphi study. As adolescents and parents/guardians were not participating as part of their work or professional role, they were offered a $AUD20 gift voucher to reimburse them for their time for completing each survey.

### Delphi consensus survey rounds

Data collection occurred between November 23, 2020 and April 1, 2021. The Round 1 survey included sociodemographic questions and 154 items to be rated by the expert panellists. The survey was completed via Qualtrics, a secure online platform for survey hosting. To complete the survey, expert panellists rated each item as ‘essential’, ‘important’, ‘don’t know/depends’, ‘unimportant’ or ‘should not be included’ regarding its importance for helping bereaved adolescents. In Round 1, free text boxes were also included at the end of each section to allow panellists to provide comments about the items or suggest additional items. These comments were read and screened for any ideas that had not already been incorporated into the survey. Ideas that were determined to be original were developed into new items and were introduced in the Round 2 survey. Items that met the criteria to be re-rated in the Round 1 survey (that is, were rated by 70–79% of the expert panel as ‘important’ or ‘essential’ as explained further in the data analysis section below) were also included in the Round 2 survey. Items that were newly added in Round 2 based on panellist feedback or met the criteria to be re-rated were included in the Round 3 survey.

### Data analysis

Data were analysed in Microsoft Excel, with panellist demographics summarised using descriptive statistics. The item ratings from the adolescent, parent/guardian and helping professional groups were merged to form one expert panel. This was to improve panel stability, to ensure that an individual panellist did not have too much endorsement weight and to prevent one panellist rating alone on deciding whether an item was endorsed or rejected [26]. The ratings of the groups were highly correlated in Round 1 (Pearson’s *r* = .83 between adolesents and parents/guardians, Pearson’s *r* = .77 between adolescents and helping professionals, and Pearson’s *r* = .83 between parents/guardians and helping professionals). Given the high consistency in endorsement ratings amongst the groups, merging these groups to form one panel was justified.

Endorsement ratings were calculated for each item by adding the percentage of panellists rating the item as ‘important’ or ‘essential’. Items that were rated as ‘essential’ or ‘important’ by 80% or more of panellists were endorsed as support that helping professionals should provide to bereaved adolescents. Items that were rated as ‘essential’ or ‘important’ by less than 80% but more than 70% of panellists were re-rated in the subsequent survey round. Items that were rated as ‘essential’ or ‘important’ by less than 70% of panellists were excluded. When re-rated in a subsequent round, items that again achieved 70–79% of ‘essential’ and ‘important’ ratings did not achieve endorsement within two rounds and were excluded. Patterns in panellists’ endorsement ratings were analysed across survey sections and in relation to more specific topics covered by separate items by comparing and contrasting endorsement rates.

### Ethics approval

This study received Human Research Ethics Committee approval from The University of Melbourne (HREC# 2057689). All participants provided informed consent before participation.

## Results

### Expert panel

The expert panel comprised a total of 49 panellists, including 16 bereaved adolescents, 14 parents/guardians, and 19 helping professionals. Panellists' characteristics are described in Table 1.

**Table 1:** Panellist characteristics (*N* = 49)

	n	% Female	Age M years (SD, range)	Time since adolescent bereavement M years (SD, range)
Adolescents	16	81.3	21.8 (3.2, 18–27)	5.5 (2.8, 2–10)
Parents/guardians	14	100.0	53.1 (3.2, 47–60)	4.6 (2.6, 0.5–9)
Helping professionals	19	79.0	50.0 (10.3, 36–70)	–

Adolescents’ relationship with the person who had died included: grandparent (*n* = 5), parent (*n* = 4), sibling (*n* = 3), close friend (n = 3), and other relative (*n* = 1). The cause of death was reported as: suicide (*n* = 8), illness/natural (*n* = 5), and accident (*n* = 2). For the parents/guardians, their adolescents’ relationship with the person who had died included: sibling (*n* = 7), parent (*n* = 3), close friend (*n* = 3), and grandparent (*n* = 1). The cause of death for this person was described as: suicide (*n* = 9), illness/natural (*n* = 2), accident (*n* = 1), homicide (*n* = 1), and unknown (*n* = 1).

Professional roles varied within the helping professional panel, including counsellors/bereavement counsellors (*n* = 6), social workers (*n* = 5), psychologists (*n* = 2), clinical team leaders/coordinators (*n* = 3), as well as one each of a case manager, psychiatrist and other allied health professional. On average, professionals had 14.7 years of experience in their professional roles, ranging from 5 to 35 years.

### Delphi consensus surveys

Table 2 presents the participation of panellists across the three survey rounds. The overall panellist retention rate quite was high, with a little over 80% of panellists who completed Round 1 also completing Round 2 and Round 3. Panellist retention was strongest amongst adolescent panellists, with 100% of adolescents who completed the Round 1 survey also completing the Round 2 and 3 surveys. Retention amongst parent/guardian panellists was also very high, with all but one completing the Round 3 survey. The lowest level of retention was observed among helping professionals, with a little less than 60% of helping professionals who completed the Round 1 survey completing all three surveys.

**Table 2:** Participation of Delphi panellists in each survey round

	Round 1 n	Round 2 n (% of Rnd 1)	Round 3 n (% of Rnd 1)
Adolescents	16	16 (100.0%)	16 (100.0%)
Parents/Guardians	14	14 (100.0%)	13 (92.9%)
Helping professionals	19	15 (78.9%)	11 (57.9%)
**Total**	**49**	**45 (91.8%)**	**40 (81.6%)**

**Fig. 1:**
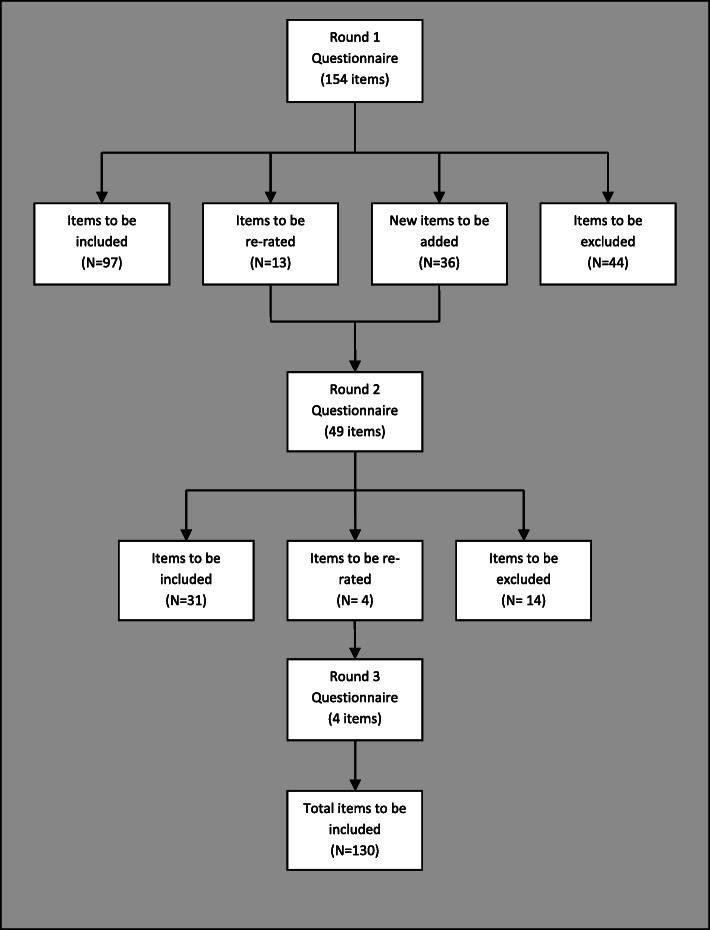
Overview of items throughout the three survey rounds

Figure 1 presents a flow chart of item ratings across the three Delphi rounds. One-hundred and ninety items were rated across the three survey rounds, resulting in 130 items (68%) endorsed as important or essential for helping bereaved adolescents and 60 items (32%) rejected. The endorsement ratings for each item across the three survey rounds are provided in Appendix B.

Table 3 summarises the number of items that were rated and endorsed in each section of the survey. All items in Section 1 about training and resources for helping professionals were strongly endorsed (endorsement ratings ranging from 90 to 100%), emphasising the importance of the helping professional having knowledge about adolescent grief as well as having undergone appropriate training to ensure they are skilled to specifically support a bereaved adolescent. The approach to providing support to the bereaved adolescent in Section 2 was also acknowledged as being important. Items about empathy, active listening, providing reassurance to the adolescent about grief being a natural and healthy process, letting the adolescent know they are supported, encouraging self-compassion, and helping the adolescent to identify their own needs, were endorsed by all panellists. Items regarding providing support at the adolescent’s pace were also strongly endorsed.

**Table 3:** Number of items rated and endorsed by section

Section	Number of items	Number (%) endorsed
1. Training and resources for helping professionals	18	18 (100%)
2. Approach to providing support	50	42 (84%)
3. Supporting bereaved adolescents	35	25 (71%)
4. Involve others in supporting the bereaved adolescent	18	6 (33%)
5. Bereavement support services for adolescents	57	29 (51%)
6. Suicide bereavement specific support	12	10 (83%)
**TOTAL**	**190**	**130 (68%)**

Endorsed items in Section 3 concerned support that encouraged adolescents to express their grief in their own way, used non-verbal and strengths-based approaches, and tailored the help to the needs and capacities of the individual adolescent. Items regarding specific therapeutic approaches (e.g., mindfulness or Cognitive Behavioural Therapy) were rejected, as well as items regarding providing grief-related information via leaflets or websites.

Section 4, about involving others in supporting the bereaved adolescent, had the lowest percentage of items endorsed. Panellists frequently gave ‘Don’t know/depends’ ratings for these items, and most items about family therapy were rejected. Items about the helping professional encouraging the adolescent to talk with others for practical and emotional support, and providing psychoeducation and support to parents to relate with their bereaved adolescents were endorsed.

Further, items with set timeframes for adolescents to be engaging with bereavement support services in Section 5 were rejected. No items were endorsed for online bereavement support, such as engaging with support via online forums, specific smart phone apps, or credible YouTube accounts. Items regarding multiple and flexible service delivery were endorsed, as well as items regarding offering services in the context of specific relationships (e.g., siblings) or cause of death.

Half of the items endorsed for providing suicide bereavement specific support in Section 6 (5 out of a total of 10 endorsed items) were developed based on panellists’ feedback provided in Round 1. The endorsed items focus on ensuring the adolescent knows that support is available shortly after being bereaved by suicide, as well as provision of long-term support, and that the helping professional openly discusses their bereavement by suicide. The items about support for parents of the adolescent bereaved by suicide were also endorsed.

## Discussion

This study aimed to determine expert consensus on recommended actions for helping professionals in providing support to bereaved adolescents, and panellists endorsed a total of 130 out of 190 statements. Overall, panellists placed a high value on helping professionals having knowledge and being skilled through appropriate training in working with bereaved adolescents. This finding is in line with previous studies that have reported that bereaved adolescents want to be supported by skilled and knowledgeable professionals, who can offer them guidance and learning opportunities to deal with their grief [15, 20, 27]. However, studies have also reported that bereaved adolescents experience important barriers (e.g., opening hours, travel distances) in accessing services and a shortage of adolescent bereavement counselors [20, 27]. Together, these findings point to a tension between the needs of bereaved adolescents and the availability of skilled adolescent grief counselors. Specialised training of adolescent grief counselors and evaluation studies of such training programs may enhance the availability of adolescent grief support [28].

Panellists strongly endorsed recommendations about helping professionals listening to bereaved adolescents and providing reassurance and empathic support. This finding is supported by research with bereaved adolescents underlining the importance of the therapeutic relationship with a counselor, being listened to, and receiving validation, which contribute to normalisation of the bereaved adolescents’ grief experiences [15, 20, 27]. The finding is also in line with research in the health and mental health field indicating the importance of the helping professionals having a collaborative approach based on respect, being non-judgemental, and open and informal engagement with the young person [29, 30]. Further research may assess the effectiveness of grief interventions based on empathic and educational approaches in this population.

Recommendations regarding strengths-based and non-verbal approaches (e.g., using creative techniques), as well as help tailored to the needs of the bereaved adolescents were highly endorsed. This is corroborated by findings from our previous research in which bereaved adolescents emphasised the importance of not being limited to engaging with a specific type of help, but rather having options they can choose at their own discretion [15, 20] Similarly, adolescents participating in broader mental health research also stressed the importance of having control about using services, and the style (e.g., verbal vs non-verbal or creative approaches) and content of the counseling [31]. Still, while these findings and the endorsed recommendations emphasise that help for bereaved adolescents must acknowledge their agency, further studies must investigate how to implement services that meet such recommendations.

Many panellists in our study rated items regarding involving others (i.e., parents or guardians) in supporting the bereaved adolescent as ‘Don’t know/depends’, indicating that the need to involve important others may vary individually depending on the needs of the adolescent and their family context. Overall, these statements were not endorsed, unlike items about providing direct support to parents of bereaved adolescents so that they are better able to help their bereaved adolescent. This finding is corroborated by our previous research revealing that bereaved adolescents preferred receiving help outside the realm of family, and usually disliked family counselling sessions [20] However, there is evidence from bereavement and mental health research that including a parental component, such as psychoeducation for parents about grief in adolescents, contributes to the effectiveness of the help provided to adolescents [25, 32–34]. Hence, the recommendation from this study to provide support to parents of bereaved adolescents is deemed appropriate to enhance the support for bereaved adolescents within their family context.

Further, panellists endorsed statements about help being provided in flexible formats and rejected statements with set timeframes for accessing professional support. This finding demonstrates the importance of the individual experience of grief and offering help and making services available both shortly after the loss as well as in the longer term. This finding is consistent with prior research, which showed that bereaved adolescents can experience a need for help at different times during their grief process [6, 35]. They can be overwhelmed by the loss or feel a need to provide support to their parents or siblings shortly after the loss rather than seeking help themselves [35, 36]. However, as bereaved adolescents mature, they may have to revisit earlier grief reactions [37]. Although such ‘regrief’ experience is a natural phenomenon, it can be very challenging and trigger a need for professional help even several years after the loss [37, 38].

Although help that is offered online or via social media could cater for the need for flexible services (which was supported by panellists in this study), statements regarding online bereavement support, such as engaging with online forums or smartphone apps, were not endorsed. These ratings demonstrate hesitancy in encouraging adolescents to access support via these platforms, and parallel findings from our previous studies in which bereaved adolescents emphasised a preference for receiving in-person support from a helping professional [15, 20]. Findings from research in the broader mental health field yield a mixed picture [30]. While some studies reported that adolescents generally do not prefer online help-seeking [39], other studies found it a useful addition to in-person counseling, especially when it was embedded in a trustworthy relationship [31]. Further, as online modes of service delivery (vs in-person) may increase due to COVID-19 restrictions, preferences for service delivery may change over time with greater experience with this modality [40, 41]. Still, it is important to note that data collection for this study did occur during the COVID-19 pandemic. Overall, the findings indicate that in-person help is more recommended to support bereaved adolescents and/or that appropriate online options for bereaved adolescents are yet to be developed [19, 42].

Panellists endorsed many statements about support specifically in the context of bereavement by suicide, showing the importance for aspects of bereavement support to be tailored for suicide bereavement. This concurs with the literature stating that although grief processes after any cause of death are similar, people bereaved by suicide, as well as helping professionals working with them, tend to emphasise that there are important differences from other forms of bereavement [10, 43]. Further, research shows that some grief experiences (e.g., feelings of guilt, rejection, struggles with “why” questions, and diminished social support) can be more pronounced in adolescent grief after suicide [17]. These features, as well as increased risks of mental health complications and suicidal behaviour in the context of loss by suicide [4, 9], may warrant suicide bereavement specific support to address suicide-specific aspects of grief and cater for the needs of this population.

The consensus recommendations that were endorsed in this study by a panel of bereaved adolescents, parents and helping professionals, may assist professionals and service providers in offering help to bereaved adolescents. The findings may also be useful in communications between adolescents, parents and helping professionals regarding how help can be provided to adolescents in the context of their particular circumstances. While the use of the recommendations does not guarantee effectiveness of the help, the recommendations may inspire development of designated adolescent grief interventions and contribute to much-needed evaluation research of such interventions [25].

### Limitations and strengths

The findings must be understood within the limitations of the study. It involved mostly female panellists, and their views may not reflect those of people who did not participate in the study. The ages of adolescent panellists ranged from 18 to 27 years, hence, recollection bias may affect the generalizability of the results to supporting younger adolescents. While there was a high correlation between the three groups in Round 1 of the study, there was an insufficient number of panellists in each of the groups to undertake comparisons. Further, the study was conducted during the COVID-19 pandemic and it is not possible to determine whether this affected the ratings of the panellists. Still, the statements included in the study were derived from the literature as well as from interview data. Panellists rated the statements, which were directly important to them, either personally or professionally, and the voice of each panellist had equal weight in the endorsements.

There was a very low attrition rate among adolescents and parents, which may indicate how important the study topic was for them and their commitment to contribute to research and clinical practice in this field. A relatively higher level of attrition was observed among helping professionals across the three survey rounds. This higher attrition rate might be attributed to the expectation that helping professionals would participate as part of their professional role (i.e., during their workday) and they may have experienced difficulties in finding time to complete the surveys. This may have been even more difficult due to an increased workload experienced amongst helping professionals during the COVID-19 pandemic. While helping professional participation in Round 3 was the lowest, this round only comprised 4 items (compared to the 154 items in Round 1) and attrition amongst helping professional panellists is therefore not expected to influence the overall findings.

## Conclusion

Involving an expert panel consisting of bereaved adolescents, parents of bereaved adolescents, and helping professionals who work with such adolescents, this study has developed consensus recommendations on how to help bereaved adolescents. The recommendations indicate that help should be offered on an ongoing basis, accessible as needed, and acknowledge the agency of the adolescent. In-person help was preferred to online help. Adequate support should be offered to parents of bereaved adolescents. Support after a loss by suicide should address suicide-related issues directly. The recommendations imply that training of helping professionals is paramount. These consensus recommendations provide a guide for helping professionals, and future implementation and evaluation studies can determine their usefulness in practice. It is anticipated that the recommendations will contribute to good practice in bereavement support as well as adolescent grief intervention studies.

## Supplementary Information

**Additional file 1: Appendix A:** Sample database search strategies.

**Additional file 2: Appendix B:** How best to provide help to bereaved adolescents: Delphi item endorsement, and rejected items.

## Data Availability

The dataset compiled and analysed during the current study are available from the corresponding author on reasonable request.
