# Correction to: How best to provide help to bereaved adolescents: a Delphi consensus study

**DOI:** 10.1186/s12888-021-03635-y

**Published:** 2022-01-07

**Authors:** Anna M. Ross, Karolina Krysinska, Debra Rickwood, Jane Pirkis, Karl Andriessen

**Affiliations:** 1 Centre for Mental Health, Melbourne School of Population and Global Health, The University of Melbourne, 207 Bouverie St., Carlton, VIC 3053 Australia; 2 Faculty of Health, University of Canberra, Bruce ACT, Canberra, 2617 Australia

**Correction to: BMC Psychiatry 21, 591 2021**

**https://doi.org/10.1186/s12888-021-03591-7**

Following the publication of the original article [1], typographic errors were identified in the Methods and Results sections.

The updated sentences in the Methods and Results sections are given below and the changes have been highlighted in **bold typeface**.

## Methods

The grey literature was searched using Google search engines of English-speaking countries **(Google.com, Google.com.au, Google.co.uk, Google.co.nz, Google.ca)**, using Google Chrome in incognito mode to avoid potential bias from the search history.

## Results

Half of the items endorsed for providing suicide **bereavement** specific support in Section 6 (5 out of a total of 10 endorsed items) were developed based on panellists’ feedback provided in Round 1.

The original article [1] has been corrected.
