# Supplementary material for: How best to provide help to bereaved adolescents: a Delphi consensus study
Source: BMC Psychiatry. 2021 Nov 23;21:591. doi: 10.1186/s12888-021-03591-7 (PMC8609510; doi:10.1186/s12888-021-03591-7)
Supplement: Supplementary file 1 — Additional file 1: Appendix A: Sample database search strategies. [file 12888_2021_3591_MOESM1_ESM.docx]

**Appendix A: Sample database search strategies**

**PsycINFO search terms**

| **#** | **Searches** |
| --- | --- |
| 16 | Limit 15 to (English language and yr=”2011- current”) |
| 15 | 12 and 13 and 14 |
| 14 | 8 or 9 or 10 or 11 |
| 13 | 4 or 5 or 6 or 7 |
| 12 | 1 or 2 or 3 |
| 11 | *Grief/ |
| 10 | grief.mp |
| 9 | bereave*.mp |
| 8 | *bereavement/ |
| 7 | youth.mp |
| 6 | teenage*.mp |
| 5 | adolescenc*.mp |
| 4 | *Adolescent/ |
| 3 | support.mp |
| 2 | help.mp |
| 1 | *Counseling/ |

Note: mp = search term, searching the following fields: title, abstract, heading word, table of contends, key concepts, original title, tests & measures, mesh; */ indicates expanding and focusing search on index search terms.

**Google search terms**

(support or help) AND (adolescen* or teen* or "young person") AND (bereave* or grief or grieving)

Ross et al. (2021). How best to provide help to bereaved adolescents: A Delphi consensus study. BMC Psychiatry.
